# Co-morbidities increase the risk of disability pension among MS patients: a population-based nationwide cohort study

**DOI:** 10.1186/1471-2377-14-117

**Published:** 2014-06-03

**Authors:** Petter Tinghög, Charlotte Björkenstam, John Carstensen, Catarina Jansson, Anna Glaser, Jan Hillert, Kristina Alexanderson

**Affiliations:** 1 Department of Clinical Neuroscience, Karolinska Institutet, Stockholm, Sweden; 2 Department of Medical and Health Sciences, Linköping University, Linköping, Sweden

**Keywords:** Multiple sclerosis, Co-morbidity, Disability pension, Sick leave, Synergistic effects, Insurance medicine

## Abstract

**Background:**

Multiple sclerosis (MS) is a chronic and often disabling disease. In 2005, 62% of the MS patients in Sweden aged 16–65 years were on disability pension. The objective of this study is to investigate whether the presence of common co-morbidities increase MS patients’ risk for disability pension.

**Methods:**

This population-based cohort study included 4 519 MS patients and 4 972 174 non-MS patients who in 2005 were aged 17–64 years, lived in Sweden, and were not on disability pension. Patients with MS were identified in the nationwide in- and outpatient registers, while four different registers were used to construct three sets of measures of musculoskeletal, mental, and cardiovascular disorders. Time-dependent proportional hazard models with a five-year follow up were performed, adjusting for socio-demographic factors.

**Results:**

All studied disorders were elevated among MS patients, regardless of type of measure used. MS patients with mental disorders had a higher risk for disability pension than MS patients with no such co-morbidities. Moreover, mental disorders had a synergistic influence on MS patients’ risk for disability pension. These findings were also confirmed when conducting sensitivity analyses. Musculoskeletal disorders appeared to increase MS patients’ risk for disability pension. The results with regard to musculoskeletal disorders’ synergistic influence on disability pension were however inconclusive. Cardiovascular co-morbidity had no significant influence on MS-patients’ risk for disability pension.

**Conclusions:**

Co-morbidities, especially mental disorders, significantly contribute to MS patients’ risk of disability pension, a finding of relevance for MS management and treatment.

## Background

Multiple sclerosis (MS) is an often progressive neurological disorder that may lead to substantial disability [1-3]. Some MS patients quickly experience permanent work incapacity while others maintain a high level of work capacity for several years [4,5]. Co-morbidity has been suggested as a key factor for understanding heterogeneity of the MS progression [6].

Research on how MS-patients are affected by co-morbidities has so far focused on other outcomes than disability pension (DP), such as ambulatory disability, [7] health-related quality of life, [8] and physical functioning [9]. It has been reported that MS patients with vascular disorders are more likely to suffer from ambulatory disability, [7] that MS patients with musculoskeletal disorders have a more rapid decline of motor functions, [10] and that mental disorders among MS patients are linked to decreased physical functioning [11] and increased perceived disability [12]. No population-based study with a comparison group of non-MS patients has, to our knowledge, been conducted to determine if MS in combination with other disorders has a synergistic influence on a disability outcome. We have chosen DP as an outcome as it also involves the social consequences of the reduced function, in terms of permanent work incapacity.

This study aimed at analyzing; 1) the presence of musculoskeletal, cardiovascular, and mental disorders in MS patients and in the general population of working ages; 2) if musculoskeletal, cardiovascular, and mental co-morbidity increase the risk of DP among MS patients; and 3) if these three types of disorders act synergistically on MS patients’ risk for DP.

This study shows that co-morbidities, especially mental disorders, significantly contribute to MS patients’ risk of disability pension.

## Methods

A population-based nationwide prospective cohort study with a five-year follow-up period (2006–2010) was conducted. All 5 709 769 people aged 17–64 who lived in Sweden in 2005 not on DP, old-age pension or with missing values on any of the covariates were followed, including all 4 519 MS patients and all those who did not receive a MS diagnosis during follow-up (N = 4 972 174), here called non-MS patients (Table 1). However, some analyses are based on all individuals with complete data and no MS-diagnosis during follow-up in order to evaluate how individuals on DP were selected with regard to socio-demographics and co-morbidities. Hence, these analyses include also those that at baseline were on early old-age pension or DP.

**Table 1:** **Baseline descriptives** (**2005**) **in percentages and incidence rates** (**IRs**) **for DP per 100 000 person**-**years** (**2006**–**2010**) **among MS patients and the general population**, **respectively**

	**MS patients**				**General population**			
	**All (n = 10 750)**	**At risk for DP (n = 4 519)**			**All (n = 5 553 120)**	**At risk for DP (n = 4 972 174)**		
	**%**	**%**	**Person-years at risk**	**IRs**	**%**	**%**	**Person-years at risk**	**IRs**
**Gender**								
Women	70.8	67.8	12 744	69.1	49.2	48.1	11 324 670	7.0
Men	29.2	32.2	6140	61.6	50.8	51.9	12 271 042	4.6
**Age (mean years)**	47.0	41.2			40.1	39.6		
**Age**-**groups**								
17-24	2.3	4.8	949	40.0	15.1	16.6	4 033 844	2.7
25-34	13.2	24.5	4 977	41.4	20.2	21.9	5 284 065	2.3
35-44	24.0	32.3	6 235	64.7	22.6	23.6	5 719 389	4.5
45-54	29.6	25.9	4 727	88.6	20.7	20.2	4 874 419	7.6
55-64	30.8	12.4	1 996	96.2	21.4	17.7	3 683 995	13.5
**Living with partner**								
No	45.1	42.1	8 018	60.6	50.3	50.1	11 733 753	5.7
Yes	54.9	57.9	10 866	71.0	49.7	49.9	11 861 959	5.6
**Educational level**								
Compulsory School (≤9 years)	17.1	9.6	1 656	92.4	20.2	18.5	4 258 677	9.2
High School (10–12 years)	49.1	45.3	8 408	75.5	47.8	47.8	11 339 322	5.9
University (≥13 years)	33.7	45.1	8 820	53.3	32.0	33.7	7 997 713	3.7
**Country of birth**								
Sweden	90.9	90.7	17 143	67.4	85.9	86.4	20 482 499	5.3
Other Nordic countries	2.9	2.4	420	81.0	3.1	2.8	621 577	10.0
EU 25	2.0	1.8	350	37.1	2.1	2.0	438 850	7.6
Other world	4.3	5.2	971	57.7	8.9	8.8	2 052 786	8.3
**Type of living area**								
Larger cities	36.8	41.6	8 137	50.8	37.1	37.5	8 854 806	4.7
Medium-sized municipalities	35.4	35.5	6 642	70.6	35.4	36.0	8 505 141	6.1
Smaller municipalities	27.8	22.9	4 105	91.6	27.5	26.5	6 235 765	6.7
**Geographic region**								
Stockholm County	22.3	26.8	5 174	46.2	21.5	21.8	3 951 156	4.6
East Middle Sweden	16.8	16.8	3 126	73.3	16.8	16.7	2 053 102	5.8
Småland and Islands	8.2	7.7	1 389	82.1	8.6	8.7	3 421 054	6.5
South Sweden	13.9	14.9	2 819	69.9	14.5	14.6	4 720 280	6.4
West Sweden	20.3	18.4	3 546	61.2	20.0	20.0	2 056 790	5.5
North Middle Sweden	8.5	7.1	1 267	90.0	8.9	8.7	934 219	5.8
Middle Norrland	3.9	3.4	607	100.5	4.0	4.0	1 302 967	7.6
Upper Norrland	6.0	5.3	956	91.0	5.6	5.5	3 951 156	7.0

### Linkage and data sources

By using the Personal Identity Number (a unique ten-digit number assigned to all Swedish residents), data from the following five nationwide registers were linked for each of the included individuals: 1) *Statistics Sweden*’*s* Longitudinal Integration Database for Health Insurance and Labor Market Studies (LISA) regarding data on socio-demographics and migration; 2) *Social Insurance Agency*’*s* database Micro Data for Analysis of the Social Insurance (MiDAS) regarding data on disability pension and diagnosis-specific sick-leave; 3) *National Board of Health and Welfare*’*s* databases National Patient Register (PAR), 4) Swedish Prescribed Drug Register (PDR), and 5) the Causes of Death Register from which data about diagnosis-specific in- and specialized outpatient care, prescribed drugs, and year of death, respectively, were obtained. All five registers are longitudinal, but differ with regard to when they were instigated. Important to mention in relation to this study is that nationwide specialized outpatient data only is available from 2001 and onwards, that reliable data on sick-leave diagnoses is available from 2004, and that the PDR register started 1 July 2005.

The study was approved by the Regional Ethical Review Board in Stockholm, Sweden.

### Outcome variable

In Sweden, all adult residents with a disease or injury that has led to permanent work incapacity are entitled to disability pension. Disability pension covers up to 64% of the lost income. The customary age for old-age pension is 65 years, but may be taken earlier. Also, all people above the age of 16 with income from work or unemployment benefits can be entitled to sickness benefits if a disease or injury has led to work incapacity.

### Exposure variable

The MS patients were identified using the nationwide PAR; that is, those who had at least one hospitalization or outpatient specialist visit due to MS as a main or secondary diagnosis during 2000–2005, classified according to the International Statistical Classification of Diseases and Related Health problems ICD-10 [13]; G35.

### Time-dependent covariates

Separate time-dependent dummy variables were constructed for musculoskeletal, cardiovascular, and mental disorders. Year-specific data for these disorders were retrieved from the PDR, MiDAS, and PAR, respectively. The first year the disorder was observed and the years following were coded as 1, while the preceding years were coded as 0. Individuals without the respective disorder were consistently coded as 0. To circumvent some of the potential drawback inherited with using register data to identify individuals with these three classes of disorders [14], three different types of measures were constructed.

In the first, and most inclusive, measure, i.e. *model 1*, individuals were classified as having musculoskeletal disorder at baseline if they had been hospitalized or received specialized outpatient care between 2000 and 2005 with a musculoskeletal disorder (ICD-10: M00-M99), or had been sickness absent due to musculoskeletal diagnoses (ICD-10: M00-M99) in 2004 or 2005. Also, from the PDR we used prescriptions for dispensed drugs licensed for musculoskeletal disorders (Anatomical Therapeutical Chemical Classification (ATC)-codes: M01-M09) in 2005. Similarly, individuals were classified as suffering from cardiovascular and mental disorders in the same manner, using the following ATC and diagnostic codes: cardiovascular disorders ATC: C01-C10; ICD-10: I00-I99 and mental disorders ATC: N05-N06; ICD-10: F00-F99.

As we were concerned about overestimation and that differential misclassification may bias the estimates obtained when applying the above described measures – in particular relevant for PDR data since no information on indication is included in this register that may make it a less specific proxy for diagnosis – two additional and more conservative measures were constructed. In the first of these, i.e. *model 2*, the classes of disorders were defined on the sole basis of the sick leave and the in- and outpatient diagnoses, i.e., PAR and MiDAS. The second type of alternative measures, i.e. *model 3*, were based on all four registers as described above (i.e. in and outpatient PAR, MiDAS and PDR), with the exception that drugs belonging to ATC groups *hypnotics and sedatives* (N05C), *centrally acting sympathomimetics* (N06BA), *anesthetics* (M01), and *muscle relaxants* (M03) were excluded, as drugs of these kinds may be prescribed to treat MS symptoms.

Cohabiting status was also constructed as a time-dependent variable, but in this case individuals were *only* classified in reference to the preceding year.

### Fixed covariates

Those living in Sweden all of 2005 were identified through LISA and the following fixed covariates, i.e. at baseline, were retrieved from LISA: age-groups (17–24, 25–34, 35–44, 45–54, 55–64); educational level [compulsory school (≤9 years), high school (10–12 years), university (≥13 years)]; country of birth (Sweden, other Nordic countries, EU 25 or other countries); type of living area [based on the H-region classification scheme [15] into the following 3 categories: larger cities (H1-H2), medium-sized municipalities (H3-H4), or smaller municipalities (H5-H6)]; and geographic region [in 8 categories; Stockholm County, South Sweden, East Middle Sweden, North Middle Sweden, Middle Norrland, Småland and Islands, West Sweden, or Upper Norrland in accordance with Eurostat’s Nomenclature of Territorial Units for Statistics, (NUTS) classification (level 2)].

### Statistical analyses

The cohort was followed from 2006 through 2010 or the year the individual turned 65, emigrated, died, or received DP, whichever came first. Hazard ratios (HRs) with 95% confidence intervals (CIs) were estimated by time-dependent proportional hazards model.

First, descriptive analyses were performed to explore the distribution of the baseline covariates among MS patients and in the general population, respectively. The absolute risks for DP by baseline characteristics were calculated for MS patients and the general population and presented as incidence rates (IRs) per 100 000 person-years.

Second, 5-year prevalence estimates, based on the three specified types of measures, of musculoskeletal, cardiovascular, and mental disorders were computed for MS patients and the general population. The MS patients’ and the general population’s 5-year prevalence estimates were compared in adjusted logistic regression analyses. Separate analyses were conducted for those at risk for DP and all individuals, i.e. including also those on early old-age pension or DP. Incidence rates for DP (IRs) per 100 000 person-years were provided for the MS patients and the general population, respectively.

Third, models based on the three specified types of measures of disorders were tested to establish whether the studied co-morbid conditions influenced the MS patients’ risk for DP. To illustrate effect-modifications also HRs with 95% CIs were calculated for the general population. Effect-modifications were evaluated using Wald ×^2^ tests.

Fourth, Rothman’s synergy index (SI) and attributable proportion due to interaction (AP) were calculated [16]. These statistics were obtained with 95% CIs, following Andersson et al’s recommendations [17]. A SI above 1 indicates a synergistic effect and a SI lower than 1 indicates an antagonistic synergistic effect. Models based on the three pre-specified types of proxy measures were tested separately.

## Results

Table 1 shows that MS patients have a different socio-demographic profile than the general population. The MS patients were more often women, cohabiting, university educated, and born in Sweden. It was also noticeable that the socio-demographic differences between MS patients and the general population became more pronounced when comparing only those at risk for DP. Furthermore, older age, lower educational level, living in a small municipality or in the northern part of Sweden seemed to be predictors of DP in the MS population. In the general population, similar trends were observed, though the absolute DP risks were overall much lower.

Table 2 reveals that the 5-year prevalence estimates of musculoskeletal, cardiovascular, and mental disorders were higher in the MS population than in the general population. This was evident in both the analyses based on the individuals at risk for DP and in analyses including *all* individuals (i.e. also those on early old-age pension or DP). In particular, it was shown that mental disorders were elevated among MS patients. All the results appeared robust, as MS patient have a significantly higher risk for all the respective disorders, regardless of the type of measure used. In general, however, the analyses based on the most inclusive of measure (model 1) rendered somewhat stronger associations.

**Table 2:** **Five year prevalence estimates for different measures** (**2000**–**2005**) **for musculoskeletal**, **cardiovascular and mental disorders among MS patients and the general population**, **with incidence rates** (**IRs**) **for DP per 100 000 person**-**years and adjusted odds ratios** (**ORs**) **with 95**% **confidence intervals** (**CIs**)

	**MS -patients**			**General Population**			**MS patients vs. General population (All)**^**d**^		**MS patients vs. General population (at risk for DP)**^**d**^	
	**All (n = 10 791)**	**At risk for DP (n = 4 519)**		**All (n = 5 618 191)**	**At risk for DP (n = 4 972 174)**					
**Model 1**^**b**^	%	%	IRs	%	%	IRs	Adj ORs	CI 95%	Adj ORs	CI 95%
**Musculoskeletal disorders**										
Yes	43.5	33.8	88.2	22.1	19.7	15.1	2.21	(2.11-2.28)	1.91	(1.79-2.03)
No			56.4			3.5				
**Cardiovascular disorders**										
Yes	24.8	15.4	90.5	14.6	12.4	15.7	1.42	(1.36-1.49)	1.34	(1.23-1.46)
No			62.6			4.4				
**Mental disorders**										
Yes	43.5	29.4	109.6	15.5	12.1	25.2	3.37	(3.24-3.50)	2.59	(2.43-2.77)
No			51.1			3.2				
**Model 2**^**a**^										
**Musculoskeletal disorders**										
Yes	22.0	18.7	81.9	15.6	13.6	17.8	1.25	(1.19-1.31)	1.37	(1.27-1.48)
No			63.3			3.9				
**Cardiovascular disorders**										
Yes	12.2	8.0	85.1	6.9	5.5	19.6	1.47	(1.39-1.57)	1.55	(1.39-1.67)
No			65.1			5.0				
**Mental disorders**										
Yes	11.7	10.3	99.8	8.9	7.1	27.7	1.20	(1.13-1.27)	1.31	(1.19-1.44)
No			62.8			4.2				
**Model 3**^**c**^										
**Musculoskeletal disorders** (exl. ATC: M01, M03)										
Yes	24.1	19.7	82.0	16.5	14.3	17.7	1.31	(1.26-1.37)	1.40	(1.30-1.50)
No			63.0			3.8				
**Mental disorders** (exl. ATC:N06AB, N05c)										
Yes	35.8	22.1	110.1	13.9	10.7	26.0	2.80	(2.69-2.91)	2.03	(1.89-2.18)
No			55.8			3.4				

^a^Prevalence estimates are based on sick-leave (MiDAS) and on in- and out-patient ICD-10 diagnoses (PAR); M00-M99, I00-I99 and F00-F99.

^b^Prevalence estimates are based on sick-leave (MiDAS) and on in- and out-patient ICD-10 diagnoses (PAR); M00-M99, I00-I99 and F00-F99, and the following ATC-codes (PDR); M01-M09. C01-C10 and N05-N06.

^c^Prevalence estimates are based on the same criteria as the model 1, except that *hypnotics and sedatives* (ATC: N05C), *centrally acting sympathomimetics* (N06BA) *anesthetics* (M01), and *muscle relaxants* (M03) are excluded from the case definition.

^d^All analyses are adjusted for gender and age-groups. In the models the general population is coded as the reference category, i.e. ORs >1 indicate that MS-patients are more likely to have a particular type of disorder.

According to model 1 (Table 3), MS patients with musculoskeletal or mental disorders had a higher risk for DP; HR 1.49 (1.33-1.67) and 2.44 (1.18-2.74), respectively. Cardiovascular disorders, however, did not appear to influence MS patients’ HR for DP; HR 1.02 (0.90-1.16). The alternative models showed similar trends; albeit musculoskeletal disorders’ influence on the MS patients’ HR for DP were weaker and non-significant in the model in which *anesthetics* (M01) and *muscle relaxants* (M03) were excluded from the case ascription definition (Model 3). Moreover, musculoskeletal, cardiovascular, and mental disorders were stronger risk factors, in relative terms, for DP in the general population than among MS patients. This is hardly surprising, given that DP overall is much more prevalent among MS patients than in the general population.

**Table 3:** **The influence of different measures for musculoskeletal**, **cardiovascular**, **and mental disorders on DP among MS patients and the general population during follow**-**up 2006**–**2010 estimated as hazard ratios (HRs) with 95**% **confidence intervals (CI)**

**Models**^**a**^	**MS patients HRs (95% CI)**	**General population HRs (95% CI)**	**Effect modifications Wald *X***^**2 **^**(p-values)**
**Model 1**^b^			
Musculoskeletal disorders	1.49 (1.33-1.67)	2.51 (2.48-2.54)	80.44 (<0.01)
Cardiovascular disorders	1.02 (0.90-1.16)	1.70 (1.68-1.72)	60.96 (<0.01)
Mental disorders	2.44 (1.18-2.74)	6.97 (6.88-7.05)	318.93 (<0.01)
**Model 2**^c^			
Musculoskeletal disorders	1.16 (1.03-1.32)	3.34 (3.30-3.71)	274.50 (<0.01)
Cardiovascular disorders	1.09 (0.92-1.29)	2.16 (2.14-2.19)	64.87 (<0.01)
Mental disorders	1.56 (1.35-1.80)	6.15 (6.08-6.22)	341.33 (<0.01)
**Model 3**^d^			
Musculoskeletal disorders	1.10 (0.98-1.53)	3.05 (3.02-3.10)	260.53 (<0.01)
Cardiovascular disorders	1.05 (0.92-1.19)	1.73 (1.71-1.75)	59.40 (<0.01)
Mental disorders	2.27 (2.03-2.53)	7.02 (6.94-7.10)	388.42 (<0.01)

^a^All models are adjusted for gender, age, educational level, country of birth, type of living area, geographic region, and cohabiting status. Cohabiting status, cardiovascular, musculoskeletal, and mental disorder are modeled as time-dependent covariates. Not having the specific disorder is the reference category.

^b^Cardiovascular, musculoskeletal, and mental disorder variables are based sick-leave (MiDAS) and in and out-patient ICD-10 diagnoses (PAR); M00-M99, I00-I99 and F00-F99, and the following ATC-codes (PDR); M01-M09, C01-C10 & N05-N06.

^c^Cardiovascular, musculoskeletal, and mental disorder variables are based on sick-leave (MiDAS) and in- and out-patient ICD-10 diagnoses (PAR); M00-M99, I00-I99 and F00-F99.

^d^Cardiovascular, musculoskeletal, and mental disorder variables are based on the same criteria as model 1, except that the drugs *hypnotics and sedatives* (ATC: N05C), *centrally acting sympathomimetics* (N06BA) *anesthetics* (M01), and *muscle relaxants* (M03) have been excluded from the case definition.

Table 4, in which the synergistic effects from model 1 are presented, shows that having mental disorders in combination with MS had a much greater influence on the HR for DP than those two disorders had individually when added up, i.e. AP 48.0% (44.1-51.8); SI 1.98 (1.84-2.14). This finding was confirmed in the model where *hypnotics and sedatives* (N05C) and *centrally acting sympathomimetics* (N06BA) drugs were excluded, and when analyses solely were based on sick-leave and in- and outpatient diagnoses, i.e. model 2 and 3 (data not shown). Musculoskeletal disorders were shown to have a synergistic influence on MS patients’ risk for DP, when the model 1 (the most inclusive model) was applied, i.e. AP 29.6% (22.3-34.9); SI 1.44 (1.33-1.56). This synergy effect was, however, not found when the more conservative case ascription methods were used, i.e. model 2 and 3 (data not shown). These additional analyses thus cast serious doubt with regard to the presence of a synergistic effect between MS and musculoskeletal disorders in relation to DP.

**Table 4:** **Musculoskeletal**, **cardiovascular**, **and mental disorders**’ **synergistic influence on DP in a five**-**year follow up**, **presented as hazard ratios** (**HRs**), **attributable proportion due to interaction** (**AP**), **and synergy index** (**SI**)^**a**^

**Models**		**HRs (95% CI)**	**AP % (95% CI)**	**SI (95% CI)**
**Model 1**	No musculoskeletal disorder and no MS	1		
	Musculoskeletal disorder (only)	3.46 (3.43-3.50)		
	MS (only)	16.74 (15.36-18.25)		
	Musculoskeletal disorder and MS	27.27 (25.36-29.34)	29.6 (22.3-34.9)	1.44 (1.33-1.56)
**Model 1**	No cardiovascular disorder and no MS	1		
	Cardiovascular disorder (only)	2.51 (2.48-2.54)		
	MS (only)	15.13 (14.18-16.15)		
	Cardiovascular disorders and MS	17.04 (15.29-18.99)	2.3 (-1-14.2)	1.03 (0.90-1.17)
**Model 1**	No mental disorder and no MS	1		
	Mental disorder (only)	8.48 (8.38-8.58)		
	MS (only)	8.66 (7.85-9.55)		
	Mental disorder and MS	30.99 (28.56-33.64)	48.0 (44.1-51.8)	1.98 (1.84-2.14)

^a^The cardiovascular, musculoskeletal, and mental disorder variables are based on sick-leave (MiDAS) and in- and out-patient ICD-10 diagnoses (PAR); M00-M99, I00-I99 and F00-F99, and the following ATC-codes (PDR); M01-M09, C01-C10 and N05-N06. Exposure variables and cohabiting status are modeled as time-dependent covariates. The models are also adjusted for gender, age, educational level, country of birth, type of living area, and geographic region.

All estimates are supplemented with 95% confidence intervals (95% CI).

Age- (16–44 and 45–64 years) and gender-stratified analyses were conducted to evaluate the fit of the models (data not shown). The estimates from these analyses (based on model 1) concerning the influence of co-morbidity were comparable across genders and age-groups. However, worth mentioning is that cardiovascular disorders were associated with a higher HR for DP among the younger MS patients, i.e. HR 1.38 (1.13-1.69).

## Discussion

This prospective and population-based register study is, as far as we know, the first dealing with how co-morbidity influences MS patients’ risk for DP. As expected, MS patients with musculoskeletal and mental co-morbidity had a higher risk for DP, but contrary to our expectation, cardiovascular disorders did not increase MS patients’ risk for DP compared to MS patients without such co-morbidity. Our results also showed that musculoskeletal, cardiovascular, and mental disorders were more common among MS patients of working ages but were, in a relative sense, stronger predictors for DP in the general population than in the MS population. Furthermore, mental disorders had a synergistic influence on MS patients’ risk for DP. The results regarding musculoskeletal disorders synergistic influence on DP were inconclusive.

The finding that musculoskeletal and mental disorders increased MS patients’ risk for DP is in accordance with previous research where different disability measures have been used [10-12]. It was, however, unexpected that cardiovascular disorders did not predict DP among MS patients. This may be interpreted as that this specific co-morbid condition is negligible in the context of MS and work incapacity, as MS in itself is a severe and disabling disorder. It may also be a result of that a cardiovascular disorder often are attained after the age of 50, when many MS patients already have experienced a reduced work capacity and been granted disability pension.

In contrast to our results, a large US cohort study found that MS patients with vascular co-morbidity at diagnosis had more than a 1.5 folded increased risk of ambulatory disability [7]. However, important differences exist; we used another outcome measure and incorporated co-morbid conditions occurring during follow-up. Moreover, the methods for defining the co-morbid disorders differed. We used four nationwide registers to identify occurrences of co-morbidity, while Marrie *et al*. [7] relied on self-reported data. Marrie *et al* used the term vascular disorders, including e.g. diabetes, while we employed ICD-10 chapters and pre-established groups of ATC-codes when defining the co-morbid disorders.

That mental disorders are highly overrepresented among MS patients has often been reported [18,19]. Several studies have also shown that the severity of MS cannot be linked to having depression or anxiety in a straight forward manner, instead they are common in all forms and stages of MS, [18,20-23] yet other studies have reported somewhat contradictory findings [11,24]. However, the majority of prior studies support the notion that the higher risk for DP among MS patients with mental disorders cannot be explained only as a consequence of especially high rates of mental disorders among severe cases of MS. Still, when interpreting the influence of mental disorders, some caution is warranted as a common pathogenic agent that influences inflammatory markers may be involved in both MS and depression [25]. and MS may sometimes cause mental disorders through purely psychological mechanisms.

In contrast to mental disorders, co-morbidity of musculoskeletal and cardiovascular disorders among MS patients has seldom been studied. Previous attempts to compare the presence of these disorders in an MS population to that in a representative population without MS have reported contradictory findings [26-28]. In the present study, all results support the notion that musculoskeletal and cardiovascular disorders are more common among individuals with MS than they are in the general population.

The strengths of the present study is its population-based and prospective cohort design, the large cohort covering a whole country, no loss to follow up, i.e. avoiding selection bias, and the use of several data sources to estimate the prevalence of co-morbidities, i.e. information about in-patient and outpatient specialized care, on specific prescribed drugs, as well as on sick-leave diagnoses – rather than self-reports. We know of no other study using such a wide spectrum of data on co-morbidity.

A potential weakness with this study concerns the potential influence of differential misclassification. First, some drugs used to operationalize musculoskeletal and mental disorders can also be prescribed for MS symptoms, e.g. hypnotics and sedatives, centrally acting sympathomimetics, anesthetics, and muscle relaxants. Second, MS patients consume more specialized health care and may thereby be more likely to become diagnosed with an additional disorder in this study. Third, it is possible that MS patients are more likely to at some point before receiving their MS diagnosis have been misdiagnosed with a musculoskeletal or mental disorder. We thus recognize that all used registers have their flaws that may both underestimate and overestimate the true differences between MS patients and the general population with regard to prevalence rates of co-morbidities. To deal with these limitations additional analyses, based on different case ascription methods were conducted. On most occasions, but not all, these analyses corroborated one another.

## Conclusions

To conclude; this study suggests that attention should be given to co-morbidity in order to better understand the DP trajectory among MS patients. This study was based on fairly broad categories of disorders and it is possible that different and/or more specific case ascriptions would nuance our findings. Additional population-based register studies focusing on how specific diagnoses or drugs influence MS patients’ work incapacity would thus be valuable.

## Competing interests

The study is supported by BiogenIdec and the Swedish Council for Working Life and Social Research. Dr. Tinghög’s work with this study is financed by a grant from BiogenIdec. Dr. Carstensen reports no competing interests. Dr. Björkenstam’s work with this study is financed by a grant from BiogenIdec. Dr. Jansson reports no competing interests. Dr. Glaser reports no competing interests. Dr. Hillert received honoraria for serving on advisory boards for BiogenIdec and Novartis and speaker’s fees from BiogenIdec, Merck-Serono, Bayer-Schering, Teva and Sanofi-Aventis. He has served as P.I. for projects sponsored by, or received unrestricted research support from, BiogenIdec, Merck-Serono, TEVA, Novartis and Bayer-Schering. His MS research is funded by the Swedish Research Council. Dr. Alexanderson received research grants from BiogenIdec.

## Authors’ contributions

PT was involved in the study design, study conception, data analysis, data interpretation, writing and editing of the manuscript. CB was involved in data interpretation and editing of the manuscript. JC was involved in data interpretation, editing of the manuscript. CJ was involved in study design, data interpretation and editing of the manuscript. AG was involved in data interpretation and editing of the manuscript. JH was involved in study design, data interpretation, and editing of the manuscript. KA was involved in the study design, study conception, data acquisition, data interpretation, writing and editing of the manuscript. All authors read and approved the final manuscript.

## Pre-publication history

The pre-publication history for this paper can be accessed here:

http://www.biomedcentral.com/1471-2377/14/117/prepub
